# Comparison between different methods of calculating Kt/V as the marker of adequacy of dialysis

**DOI:** 10.12669/pjms.38.1.4281

**Published:** 2022

**Authors:** Fazl-e-Mateen, Sajad Ahmad, Irfan Elahi, Muhammad Anees

**Affiliations:** 1 Dr. Fazl-e-Mateen, FCPS. Assistant Professor, Department of Nephrology, Mayo Hospital, King Edward Medical College, Lahore, Pakistan; 2 Dr. Sajad Ahmad, FCPS. Assistant Professor, Department of Nephrology, Mayo Hospital, King Edward Medical College, Lahore, Pakistan; 3 Dr. Irfan Elahi, FCPS Assistant Professor, Department of Nephrology, Mayo Hospital, King Edward Medical College, Lahore, Pakistan; 4 Prof. Muhammad Anees, FCPS Professor/ Head of Department, Department of Nephrology, Mayo Hospital, King Edward Medical College, Lahore, Pakistan

**Keywords:** Hemodialysis, Kt/V, Online clearance monitoring, Daugirdas Formula

## Abstract

**Objectives:**

To compare different methods of calculating adequacy of hemodialysis in terms of Kt/V.

**Methods:**

This was an observational, quantitative study undertaken after the approval of Internal Review Board at the Hemodialysis Unit of Nephrology department, Mayo Hospital, Lahore from 1st December 2018 to 30th June 2019. Sixty hemodynamically stable patients of end stage renal disease undergoing hemodialysis for more than three months with age 18 to 70 years were included in the study by convenience non probability sampling. Critically ill patients with multiple co-morbidities like sepsis (i.e. total leukocyte count >11000 or <4000 x 109/L), ischemic heart disease, pace- makers, malignancies, cirrhosis etc. were excluded. Patients who were not adherent to dialysis prescription or hemodynamically unstable were also excluded. One way ANOVA and Pearson’s correlation were used to find correlation between three methods of measuring Kt/V.

**Results:**

Mean ultrafiltration was 2.1+ 0.76 liter/session. Pre dialysis weight was 64.7 +14.7 kgs, mean post dialysis weight was 62.5 + 14.7 kgs. For every patient blood flow rate was 300 ml/m and dialysis flow rate was 500 ml/min. The mean values of Kt/V measured by Daugirdas formula was 1.35 ± 0.33, mean online clearance monitoring (OCM) value was 1.17 ± 0.29 and by normogram was 1.36 ± 0.33. There was positive significant correlation between values of Daugirdas formula, Normogram and online clearance monitoring (OCM) i.e. r = 0.897 (p-value < 0.001) measured by Pearson’s correlation and one way-ANOVA.

**Conclusion:**

Online clearance monitoring can be used for measuring adequacy of hemodialysis, but OCM slightly underestimates Kt/V as compared to Daugirdas formula and Normogram.

## INTRODUCTION

Hemodialysis (HD) is the most widely used form of renal replacement therapy in Pakistan.^1^ The mortality and morbidity of patients on hemodialysis depends upon variable factors like pre-existing conditions like Diabetes Mellitus, Hypertension, Ischemic heart disease, nutritional status of patient and adequacy of dialysis.^2^ Adequacy of dialysis is found to be the most important factor contributing in mortality and morbidity of hemodialysis patients. Adequacy of dialysis is assessed by different methods including on line and through blood sample analyzed. Adequacy of dialysis is conventionally measured by urea reduction ratio (URR) and Kt/V (K is the Urea clearance, *t* is time of dialysis and V is volume of distribution of patient).^3^ Kidney Disease Initiative Global Outcome KDIGO guidelines have advocated for target Kt/V of 1.4 per session.^4^

There are three methods of calculating Kt/V which include estimation by Daugirdas Formula, online clearance monitoring (OCM) and via normogram.^5^ OCM provides noninvasive method to measure Kt/V because there are no blood sampling involved. On-line clearance monitors measure the difference in conductivity between the dialysate entering and leaving the dialyzer with two different dialysate electrolyte concentration monitors. These measurements give ionic dialysance of sodium ion, which is equivalent to effective urea clearance.^6^ According to some studies OCM results underestimate the efficiency of the dialysis as compared to the calculated Kt/V values.^7^ The use of OCM gives opportunity to measure Kt/V for every session without any blood sampling. In previous studies there was significant correlation between Kt/V measured by OCM and Daugirdas formula, but those studies did not include normogram.

Estimation by URR, Normogram and Daugirdas formula involves repeated blood sampling and investigations. But there is little locally available data whether OCM can be used in place of URR and Kt/V by Daugirdaus formula. This study was conducted to establish the correlation and role of OCM in regular monitoring of Kt/V.

## METHODS

This was an observational, quantitative study undertaken after the approval of Internal Review Board at the Hemodialysis Unit of Nephrology department, Mayo Hospital, Lahore from 1st December 2018 to 30th June 2019. Sixty hemodynamically stable patients of end stage renal disease undergoing hemodialysis for more than three months with age 18 to 70 years of either gender were included in the study by convenience non probability sampling. Informed consent was taken from all patients. Critically ill patients with multiple co-morbidities like sepsis (i.e. total leukocyte count >11000 or <4000 x 109/L), ischemic heart disease, pace- makers, malignancies, cirrhosis, etc. were excluded. Patients who were not adherent to dialysis prescription or hemodynamically unstable were also excluded.

Hemodialysis were done on Fresenius 4008S hemodialysis machines with 1.8 m^2^ dialyzer. Duration of dialysis was four hours per session. In this cross- sectional study, adequacy of hemodialysis of the recruited patients was estimated by comparing calculated Kt/V by Daugirdas formula, Normogram, online clearance monitor and urea reduction ratio. Each patient that underwent two blood sampling. First sample was taken at the initiation of the hemodialysis session and the other sample to be obtained at the end of the same session after setting ultra- filtration rate to zero, reducing blood pump flow to less than 100 ml/min for 10-20 seconds and then stopping pump flow. At this point, blood sample was obtained from arterial blood line sampling port and both these labeled samples were sent to the pathology laboratory of Mayo Hospital for the measurement of serum Urea, Creatinine, Bicarbonate, Sodium and Potassium levels.

### Ethical Approval:

(Ref: 278/RC/KEMU, Dated: 29-11-2018).

Following formulas were used to measure Kt/V and URR:

Daugirdas formula: Kt/Vsp = -ln (R – 0.008 x t) + (4 – 3.5 x R) x Uf/W8

(ln: natural logarithm, R: ratio of postdialytic ÷ predialytic BUN, t: effective dialysis time in hours, Uf: ultrafiltration volume in litres, W: weight of the patient after dialysis in kg. Kt/Vsp: Single pool Kt/V)

Urea reduction ratio URR: Pre-dialysis Urea – Post-dialysis Urea/Pre-dialysis Urea

Data was entered and analyzed by IBM SPSS version 20.0. Mean ± SD was calculated for quantitative data like age, Kt/V and duration of dialysis. Frequency and percentages was calculated for qualitative data like gender. One way-ANOVA and Pearson correlation were used to find the correlation between Daugirdas formula, Normogram and OCM. A p-value ≤0.05 as significant.

## RESULTS

The mean age of all patients was 42.92 ± 13.07 years. 40% patients were less than 40 years of age and 60% patients were more than 40 years. There were 40 (66%) male and 20(34%) female cases. A total of 21(35%) cases had duration of disease for 3-6 months and 39(65%) cases had duration of disease > 6 months. According to access type, 56 (93.3%) cases had AV fistula, 2(3.3%) cases had double lumen catheter and 2(3.33%) cases had AV graft.

**Fig.1:**
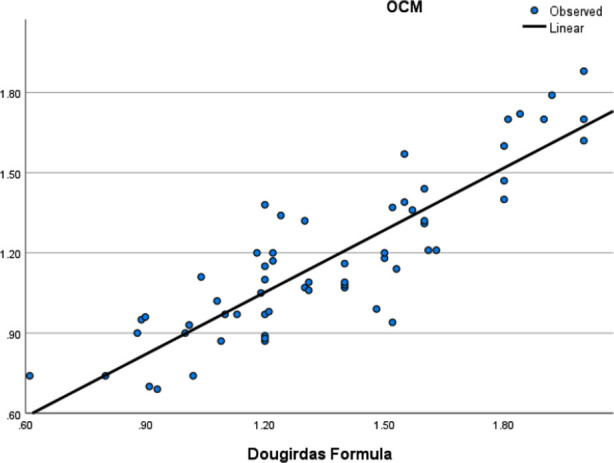
Graph showing linear regression between Kt/V by Daugirdas formula and Kt/V by OCM.

**Fig.2:**
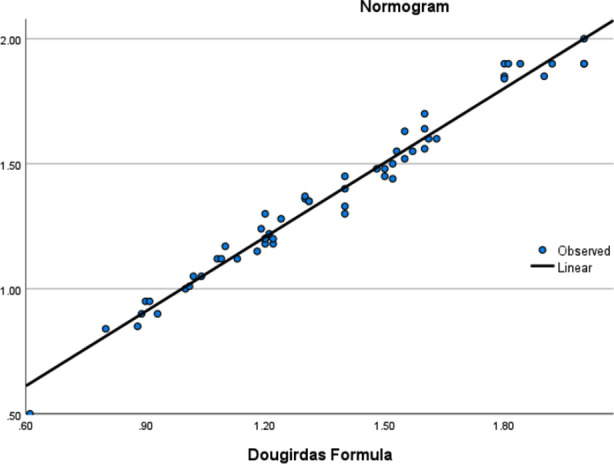
Graph showing linear regression between Kt/V by Daugirdas formula and Kt/V by Normogram.

Mean ultrafiltration was 2.1+ 0.76 liter/session. Pre dialysis weight was 64.7 +14.7 kgs, mean post dialysis weight was 62.5 + 14.7 kgs. For every patient blood flow rate was 300 ml/m and dialysis flow rate was 500 ml/min. Laboratory investigations of each patient are shown in Table-I. The mean values of Kt/V measured by Daugirdas formula was 1.35 ± 0.33, mean OCM value was 1.17 ± 0.29 and by normogram was 1.36 ± 0.33 (Table-II). There was no significant difference between Daugirdas formula and OCM by ANOVA (p=0.995). Though there is statistically significant difference between OCM and Daugirdas formula shown in Table-II by ANOVA, still significant correlation was found between two methods. There was positive significant correlation of Daugirdas formula with Normogram and OCM i.e. r = 0.998 and r = 0.874 respectively (p-value < 0.001) measured by Pearson’s correlation.Fig.1 & 2 shows positive correlation on linear regression model of Daugirdas formula with OCM and Normogram repecively.

**Table I:** Table showing baseline characteristics of patients (n=60).

	Mean
Ultrafiltration (L)	2.18± .97
Pre-dialysis Weight (kgs)	64.75± 14.76
Post-dialysis Weight (kgs)	62.51± 14.72
QB (ml/min)	300±0.0
QD (ml/min)	500±0.0
Pre-dialysis Urea (mg/dl)	149.62± 40.8
Post-dialysis Urea (mg/dl)	47.75± 17.30
HCO3	14.72 ±1.79
Na+	135.65 ±2.97
K+	4.2±.48

**Table II:** Kt/V values measured by different methods.

Method	Mean Kt/V	p value ANOVA
Daugirdas Formula	1.35±o.33	DF vs. Norm = 0.995 DF vs. OCM = 0.005* Norm vs. OCM = 0.004*
OCM	1.17±0.29	
Normogram	1.36±0.3	

DF: Daugirdas Formula, Norm: Normogram.

**Table III:** Correlation between Kt/V values measured by different methods.

	Correlation coefficient (r) Daugirdas Formula	p value Pearson’s correlation
OCM	0.874	< 0.001*
Normogram	0.998	< 0.001*

Majority of the patients had Arteriovenous fistula 93.3%, 3.3% had AV graft and 3.3% had double lumen catheters. When type of AV access were correlated with Kt/V measured by OCM and Daugirdas formula by pearson correlation, there was no significant correlation found between type of AV access and Kt/V (p> 0.5).

## DISCUSSION

In this study most patients were men (40/60). This is in comparison with a study done by Breitsamter et al where males were 56%.^9^ Most common underlying disorder was Diabetes Mellitus (53.3%) which is in agreement with a study done by Baloglu et al.^10^ There was no significant relation of type of AV access with Kt/V. As Blood flow, dialysate flow and duration of session were constant for all patients so their effect on Kt/V couldn’t be studied.

In this study efficacy of hemodialysis was measured by three methods. In most studies Kt/V was measured by two methods only i.e., Daurgirdas (Kt/V_D_) and OCM(Kt/V_OCM_). In OCM ‘V’ of Kt/V was measured by Watson formula which is default for Fresenius hemodialysis machines. While some studies also used other methods to measure Volume of distribution like Bioimpedence method.^11^

Mean Kt/V measured by Daugirdas formula was 1.36 while mean Kt/V_OCM_ was 1.17. In our study OCM slightly underestimated the Kt/V as compared to Daugirdas formula. This has been shown by many studies.^5^,^9^,^12^ A local study done at C.M.H. Peshawar also showed the same results, but showed positive correlation among two methods.^13^ The reason for this slight underestimation has been discussed by few investigators. Lindley has shown that ‘V’ measured by Watson formula underestimates Kt/V as compared to ‘V’ measured by bioimpedence. This is the reason our study showed mean Kt/V_OCM_ less than Kt/V_D_. On the other hand, Kt/V by Daugirdas correlate more closely wth Kt/V by normogram (Kt/V_N_), because in normogram URR is also required which is measured by blood samples and, values of ultrafiltration and post-dialysis weight are also used like Daugirdas formula. There was significant difference shown by ANOVA between OCM and Daugirdas which was also seen in a study by Breitsameter et al.^9^ However, correlation between all three methods were statistically significant (P=0.000) measured by Pearson’s correlation, and linear regression model. Many studies have shown though OCM slightly underestimate Kt/V but it is a simple and reliable method to monitor Kt/V.^9^,^12^,^13^ As there are no blood sampling involved Kt/V can be measured for every dialysis session. Our mean Kt/V was also accordance with KDIGO guidelines which recommend Kt/V of 1.4 for each session.

In a Spanish study done by Teruel JL Kt/V was also measured by LOWIRE’s formula and compared with Daugirdas formula.^14^ Study showed Daugirdas formula overestimated Kt/V as compared to Lowrie’s formula. It was postulated that Daugirdas formula measures convective losses and OCM on the other hand measures diffusive losses only. This is the reason Kt/V measured by Daugirdas is slightly higher than Lowrie’s and OCM.

Normogram was also used in this study which involve measurement of URR by blood sampling, ultrafiltration volume and post dialectics weight. These values are plotted on a specially designed normogram which gives value of Kt/V. In our study Kt/V measured by Normogram is more comparable to Daugirdas formula. Its use is limited because it also involves measurement of pre- and post-dialysis urea, ultrafiltration volume and post dialytic weight. It is cumbersome to plot these values on a graph, while in Daugirdas formula these values need to be put in online calculators which calculate value for Kt/V.

### Limitations:

First it was a single center study. To validate the use of OCM as a measuring tool we need to conduct this indifferent centers and on different population groups. Secondly sample size was less, further studies with higher number of patients are needed to validate our results. Thirdly all hemodialysis machine used are of same brand and model, this OCM model should be checked on different brands of hemodialysis machines so it can be implemented across all over country.

## CONCLUSION

Online Clearance Monitoring can be used as noninvasive tool for measuring dialysis adequacy, however, it should be kept in mind that OCM slightly underestimates Kt/V as compared to Daugirdas formula and Normogram.

### Authors’ Contribution:

**FM:** Conceived, designed and did statistical analysis & editing of manuscript. Accuracy or integrity of the work.

**FM & SA:** Did data collection and review of literature.

**IE:** Did data collection and writing of manuscript.

**MA:** Did review and final approval of manuscript.

## References

[1] Janjua TK, Mukhtar KN, Naveed AK, Ahmed EB, Rehan M (2019). Frequency of maintenance hemodialysis patients meeting K/DOQI criteria for serum calcium, phosphorus, calcium phosphorus product and PTH levels;a single institutional experience from Pakistan:A cross sectional study. Pan Afr Med J.

[2] Miskulin D, Bragg-Gresham J, Gillespie BW, Tentori F, Pisoni RL, Tighiouart H (2009). Key comorbid conditions that are predictive of survival among hemodialysis patients. Clin J Am Soc Nephrol.

[3] Barzegar H, Moosazadeh M, Jafari H, Esmaeili R (2016). Evaluation of dialysis adequacy in hemodialysis patients:A systematic review. Urol J.

[4] National Kidney Foundation (2015). KDOQI Clinical Practice Guideline for Hemodialysis Adequacy:2015 update. Am J Kidney Dis.

[5] Aatif T, Hassani K, Alayoud A, Zajjari Y, Maoujoud O, Benyahia M (2014). Quantification of hemodialysis dose:what Kt/V ?to choose?. Int J Artif Organs.

[6] Alayoud A, Montassir D, Hamzi A, Zajjari Y, Bahadi A, Kabbaj DE (2012). The Kt/V by ionic dialysance:Interpretation limits. Indian J Nephrol.

[7] Al Saran K, Sabry A, Abdulghafour M, Yehia A (2010). Online conductivity monitoring of dialysis adequacy versus Kt/V derived from urea reduction ratio:A prospective study from a Saudi Center. Ren Fail.

[8] Sternby J, Daugirdas JT (2015). Theoretical basis for and improvement of Daugirdas'second generation formula for single-pool Kt/V. Int J Artif Organs.

[9] Breitsameter G, Figueiredo AE, Kochhann DS (2012). Calculation of Kt/V in haemodialysis:A comparison between the formulas. J Bras Nefrol.

[10] Baloğlu İ, Selçuk NY, Evran H, Tonbul HZ, Turkmen K (2019). Evaluation of Hemodialysis Adequacy:Correlation between Kt/Vurea and Other Methods. Turk J Nephrol.

[11] Martinez Fernandez G, Ortega Cerrato A, Masia Mondejar J, Perez Rodriguez A, Llamas Fuentes F, Gomez Roldan C (2013). Efficacy of dialysis in peritoneal dialysis:utility of bioimpedance to calculate Kt/V and the search for a target Kt. Clin Exp Nephrol.

[12] Lindley EJ, Chamney PW, Wuepper A, Ingles H, Tattersall JE, Will EJ (2009). A comparison of methods for determining urea distribution volume for routine use in on-line monitoring of haemodialysis adequacy. Nephrol Dial Transplant.

[13] Begum M, Arshad AR (2020). Comparison of Kt/V Estimation by Online Clearance Monitoring and Daugridas Formula.

[14] Teruel JL, Fernandez Lucas M, Arambarri M, Merino JL, Echarri R, Alarcón C, Marcen R, Rivera M, Ortuno J (2003). Utilidad de la dialisancia ionica para control de la dosis de dialisis. Experiencia de un ano [Ionic dialysance to control the dose of dialysis. One year experience]. Nefrologia.

